# Supplementary material for: TLR9 Monotherapy in Immune-Competent Mice Suppresses Orthotopic Prostate Tumor Development
Source: Cells. 2024 Jan 2;13(1):97. doi: 10.3390/cells13010097 (PMC10778079; doi:10.3390/cells13010097)
Supplement: Supplementary file 1 [file cells-13-00097-s001.zip › Miles et al (Cells) Supplementary figures.pdf]

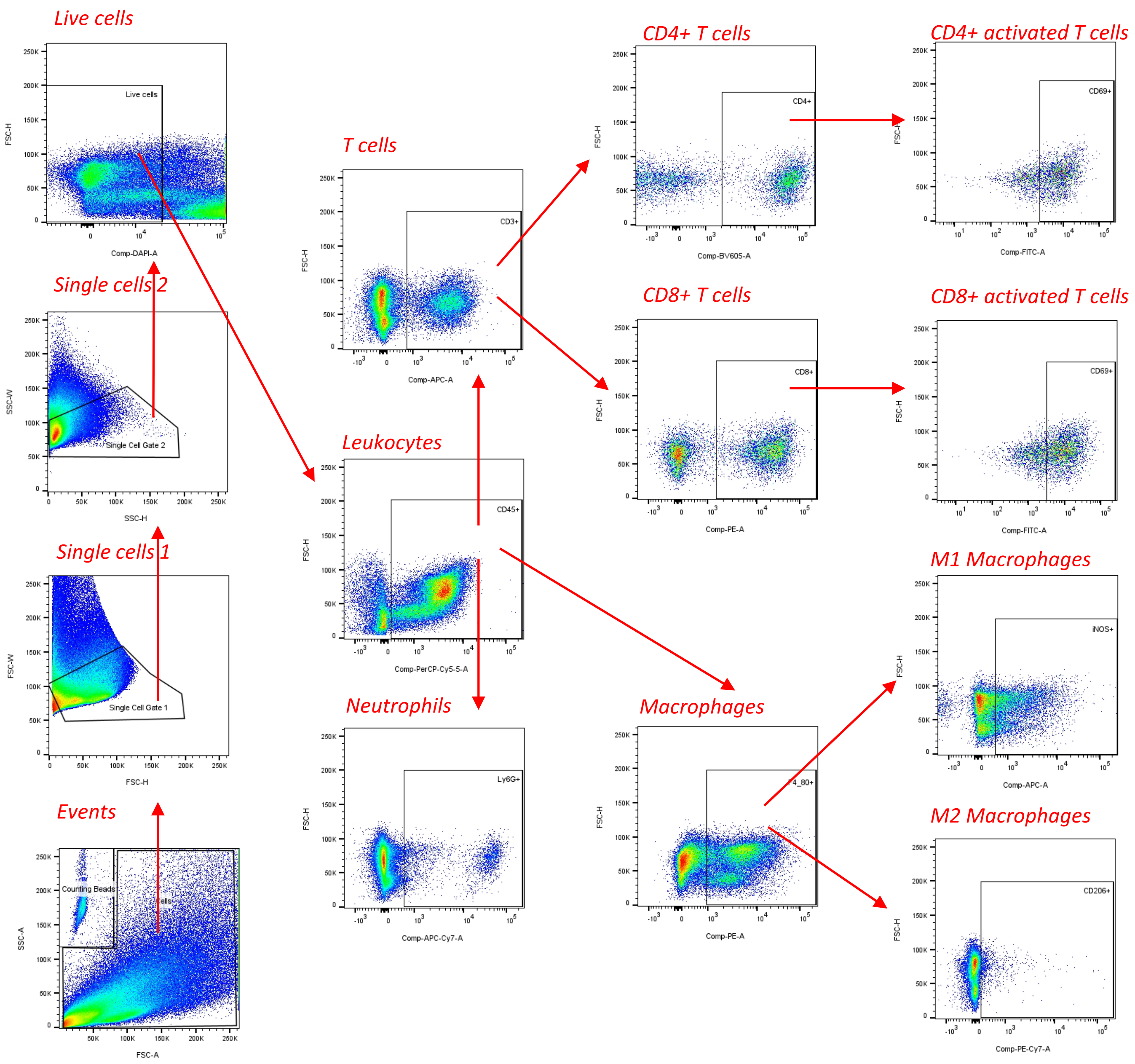

Figure S1. Flow cytometry gating strategy.

CD45+ leukocytes were gated from live cells and divided into CD3+ T cells, F4/80+ macrophages or Ly6G+ neutrophils. T cells were further subdivided into CD4+ (helper) or CD8+ (cytotoxic) T cells, and CD69+ co-staining performed to measure “activated” T cell subtypes. Macrophages were further subdivided into iNOS+ (M1) or CD206+ (M2) macrophages.

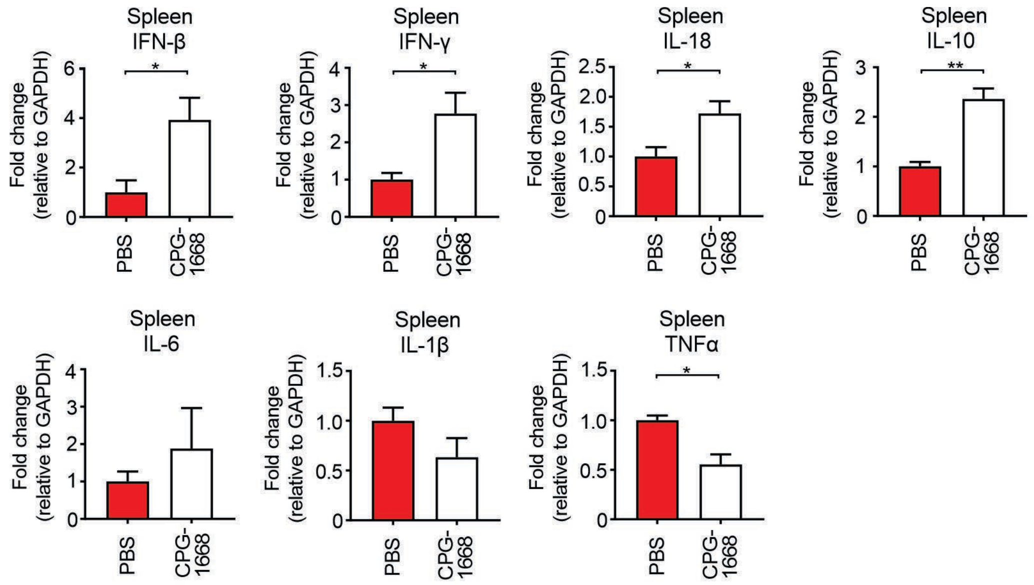

Figure S2. CPG-1668 administration increases a subset of inflammatory genes in the spleen.

C57BL6/J mice were treated with PBS or CPG-1668 (50 µg/day) and spleens harvested after 3 days. QPCR analysis of various inflammatory genes was performed. Data is presented relative to GAPDH housekeeping and expressed relative to PBS controls. Data represent n = 3 per group and expressed as mean ± SEM. Statistical analysis was conducted using an unpaired, two-sided t-test (* $p<0.05$).

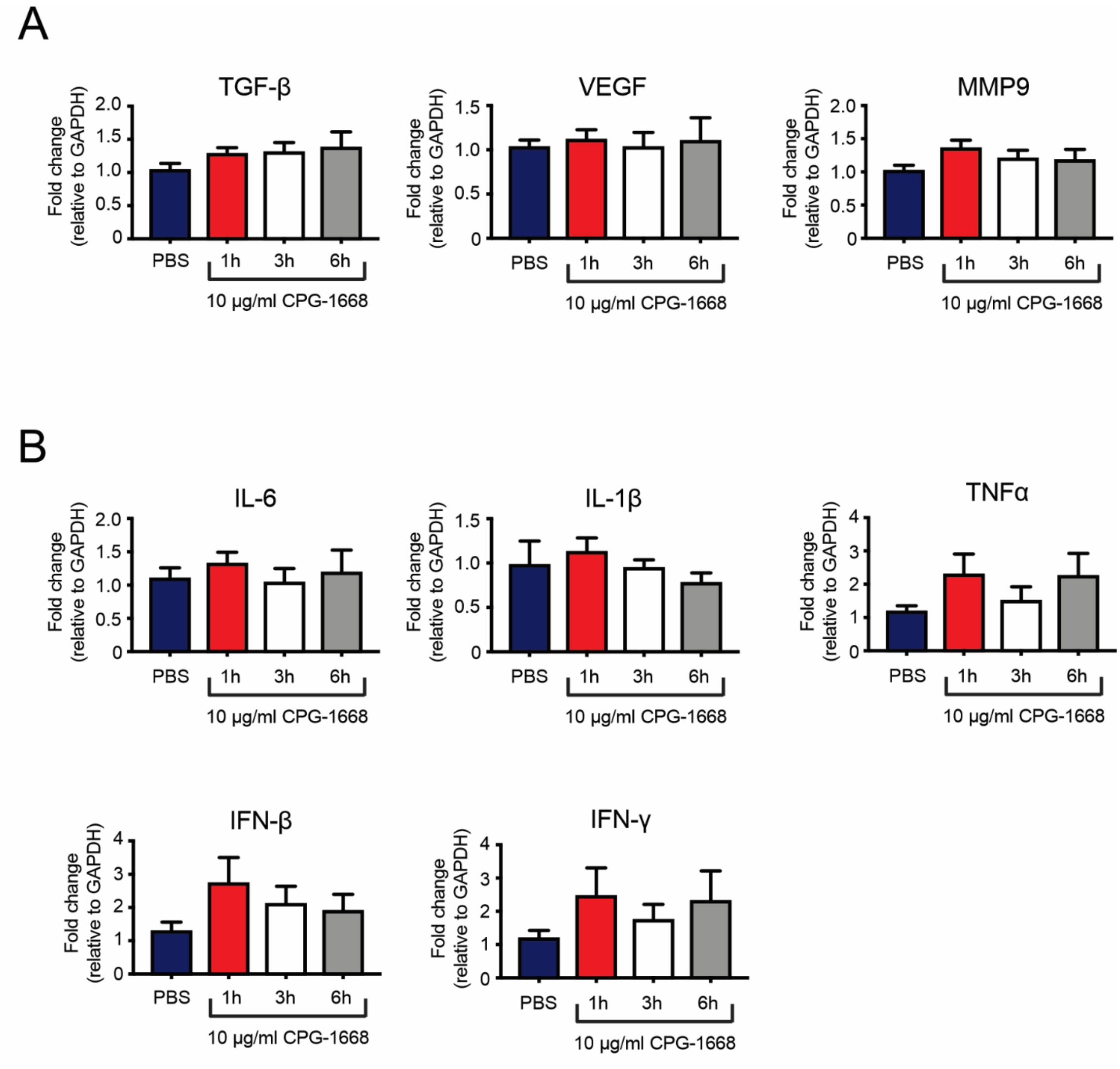

Figure S3. TLR9 activation has no effect on tumour promoting or inflammatory markers in RM1 murine prostate cancer cells at early timepoints.
RM1 prostate cancer cells were treated *in vitro* with PBS or 10 µg/ml CPG-1668 for 1, 3 or 6 hours. QPCR analysis of (**A**) tumour promoting genes (MMP9, TGF-ß and VEGF) or (**B**) inflammatory genes (IL-6, IL-1β, TNFα, IFN-β and IFN-γ) was performed. Data is presented relative to GAPDH housekeeping and expressed relative to PBS controls. Data represent n = 12-14 per group and expressed as mean ± SEM. Statistical analysis was conducted using one-way ANOVA followed by Dunnett's post hoc test for multiple comparisons (statistical significance for all conditions was $p>0.05$).

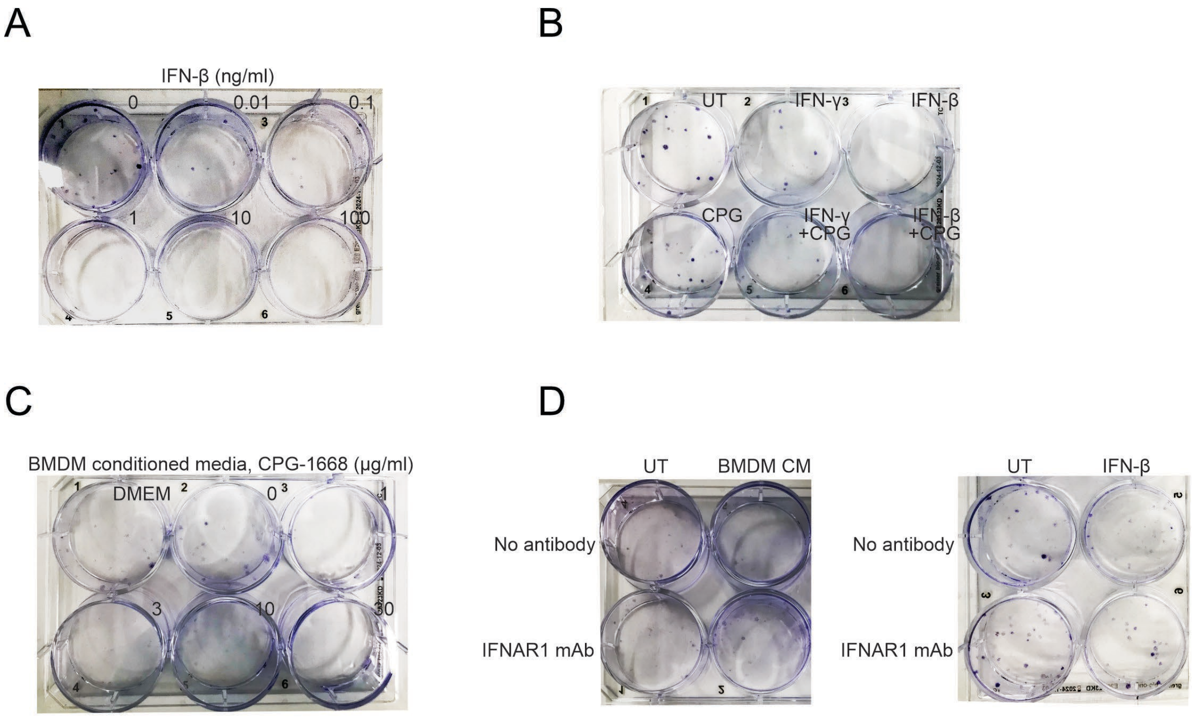

Figure S4. Representative images of colony forming assays.
RM1 prostate cancer cells were exposed to the following conditions for 24 hours: (**A**) increasing concentrations of recombinant IFN-β, (**B**) CPG-1668 (10 µg/mL), IFN-γ (100 ng/mL) or IFN-β (100 ng/ml) alone or in combination, (**C**) conditioned media (CM) from bone marrow-derived macrophages (BMDM) grown in increasing concentrations of CPG-1668 for 24 hours, or (**D**) pretreated or not with 50 µg/mL IFNAR1 blocking monoclonal antibody for 1 hour before the addition of BMDM-derived CM or recombinant IFN-β (1 ng/mL). After exposure, cells were seeded in 6-well plates in complete media then stained with crystal violet after 7-8 days.

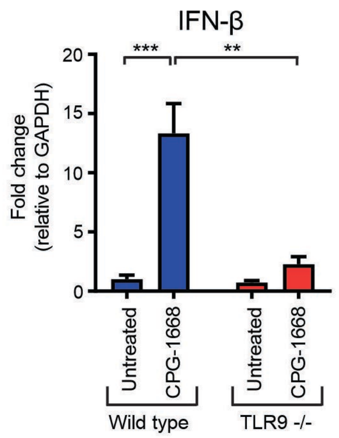

Figure S5. CPG-1668 induces IFN-β upregulation in macrophages via TLR9.
Wild type or TLR9-deficient (TLR9 -/-) bone marrow-derived macrophages were treated *in vitro* with 1 µg/ml CPG-1668 or not 24 hours, and IFN-β gene expression was performed. Data is presented relative to GAPDH housekeeping and expressed relative to untreated wild type controls. Data represent n = 3 per group and expressed as mean ± SEM. Statistical analysis was conducted using two-way ANOVA followed by Tukey's post hoc test for multiple comparisons (** $p<0.01$ *** $p<0.001$).
